# Accuracy of patient setup positioning using surface‐guided radiotherapy with deformable registration in cases of surface deformation

**DOI:** 10.1002/acm2.13493

**Published:** 2022-01-25

**Authors:** Boriphat Kadman, Akihiro Takemura, Tatsuya Ito, Naoki Okada, Hironori Kojima, Shinichi Ueda

**Affiliations:** ^1^ Division of Health Sciences Graduate School of Medical Sciences, Pharmaceutical and Health Sciences Kanazawa University 5‐11‐80 Kodatsuno Kanazawa Ishikawa 9200942 Japan; ^2^ Faculty of Health Sciences Institute of Medical, Pharmaceutical and Health Sciences Kanazawa University 5‐11‐80 Kodatsuno Kanazawa Ishikawa 9200942 Japan; ^3^ Department of Radiological Technology Japanese Red Cross Aichi Medical Center Nagoya Daini Hospital 2‐9 Myouke‐cho, Showa‐ku Nagoya Aichi 4668650 Japan; ^4^ Department of Radiology Kanazawa University Hospital 13‐1 Takara‐machi Kanazawa Ishikawa 9208641 Japan

**Keywords:** breast deformation, mean deviation setup error, deformable algorithm, surface‐guided radiotherapy

## Abstract

The Catalyst™ HD (C‐RAD Positioning AB, Uppsala, Sweden) is surface‐guided radiotherapy (SGRT) equipment that adopts a deformable model. The challenge in applying the SGRT system is accurately correcting the setup error using a deformable model when the body of the patient is deformed. This study evaluated the effect of breast deformation on the accuracy of the setup correction of the SGRT system. Physical breast phantoms were used to investigate the relationship between the mean deviation setup error obtained from the SGRT system and the breast deformation. Physical breast phantoms were used to simulate extension and shrinkage deformation (−30 to 30 mm) by changing breast pieces. Three‐dimensional (3D) Slicer software was used to evaluate the deformation. The maximum deformations in *X*, *Y*, and *Z* directions were obtained as the differences between the original and deformed breasts. We collected the mean deviation setup error from the SGRT system by replacing the original breast part with the deformed breast part. The mean absolute difference of lateral, longitudinal, vertical, pitch, roll, and yaw, between the rigid and deformable registrations was 2.4 ± 1.7 mm, 1.3 ± 1.2 mm, 6.4 ± 5.2 mm, 2.5° ± 2.5°, 2.2° ± 2.4°, and 1.0° ± 1.0°, respectively. Deformation in the *Y* direction had the best correlation with the mean deviation translation error (*R* = 0.949) and rotation error (*R* = 0.832). As the magnitude of breast deformation increased, both mean deviation setup errors increased, and there was greater error in translation than in rotation. Large deformation of the breast surface affects the setup correction. Deformation in the *Y* direction most affects translation and rotation errors.

## 1 INTRODUCTION

Correction of the patient positioning setup in radiotherapy treatment improves the delivery of a high dose to the target volume. Traditional radiotherapy treatment places marks on the skin for laser‐based setup that correlate with the radiation imaging.^1^ Markers, pens, and henna are used for marking in a manner that is non‐invasive, easy, and painless to the patient, but are uncomfortable and cosmetically of a concern to the patient. Meanwhile, the technique of tattooing injects a needle into the patient's skin at a few points. This invasive technique of marking the skin is convenient to the radiographer but painful to the patient.^2^ Nowadays, in‐room imaging technology has the potential to provide an accurate positioning setup. Image‐guided radiotherapy (IGRT) is a modern radiotherapy technique that uses ionizing and non‐ionizing radiation systems. The standard method of IGRT is cone‐beam computed tomography (CBCT) using anatomical landmarks. A non‐ionizing radiation camera‐based or optical tracking system is used to identify the setup point without additional radiation and thus reduce the radiation dose in the setup positioning and online motion monitoring during treatment.^1^, ^2^, ^3^ Such surface‐guided radiotherapy (SGRT) systems verify and correct the positioning using the skin surface of the patient.

An example of commercial equipment used in SGRT is Catalyst™ HD (C‐RAD Positioning AB, Uppsala, Sweden), which adopts a deformable model.^4^ The Catalyst™ HD system can correct the setup positioning and detect the deviation of the position of the patient before treatment from the position in the computed tomography (CT) simulation. Reference data are obtained by a treatment planning system and imported to the Catalyst™ HD system for comparison with the actual position. The Catalyst scanner scans and creates a live image. The operation of surface matching adopts a deformable algorithm to match the reference image and live image. The results of correction are then calculated as absolute and relative corrections. Additionally, the Catalyst™ HD system corrects the posture error to adjust the extremity and chin positions relating to the treatment area; for example, the shoulder, arm, and chin positions for breast cancer. The system adopts a modified deformable iterative closest point (ICP) algorithm to create a deformable node graph from the reference surface. The algorithm finds point‐by‐point correspondence between target and reference surfaces and applies a transformation to the reference surface.^5^, ^6^, ^7^ The Catalyst™ HD system is used in the treatment of all cancer regions, including the head, thorax, abdomen, and extremities.^4^ In particular, the optical surface system is widely used in radiotherapy for breast cancer patients.

Breast‐conserving surgery (BCS) followed by radiation therapy is the standard treatment for early breast cancer. However, the clinical effect of a course of radiotherapy includes the acute or late toxicity of a high dose for normal tissue; examples of effects are changes in texture and color of the irradiated area, fibrosis, breast shrinkage, osteoporosis, and pulmonary problems.^8^ Breast edema affects the breast shape in cancer patients. Additionally, breast deformation contributes to rotational errors in the setup positioning in all three directions. Breast deformation reportedly occurs for women having undergone BCS and affects the dose distribution, with large deformations potentially resulting in the underdosing of the target volume.^9^, ^10^, ^11^ In the case of surface deformation, the deformable image registration algorithm of the Catalyst™ HD system is used by adopting a deformable reference mesh based on the ICP algorithm for each node, and the mesh is fitted to a deformable model and the surface shape is reconstructed.^7^, ^12^ The challenge in using the Catalyst™ HD system is accurately correcting the positioning setup when the body of the patient has deformed. This study evaluates the effect of breast deformation on the accuracy of a surface imaging system.

## 2 MATERIALS AND METHODS

### 2.1 SGRT system

The Catalyst™ HD is used for SGRT. In clinical use, this system assists in adjusting the positioning of the patient during setup, monitors the positioning of the patient, and assists with gating and deep inspiration breath‐hold (DIBH) during treatment. The SGRT system has three cameras oriented at intervals of 120°. Near‐invisible violet patterns are projected as a color map representing posture error onto the surface of the patient and measured. The patterns are captured by the three cameras and a model of the external surface of the patient is reconstructed. The software interfaces to the program of the linear accelerator and gives the direction of positioning correction toward the reference setup. This system does not require temporary marks or permanent tattoos on the patient's skin.^4^, ^13^, ^14^ The algorithm of the SGRT system adopting non‐rigid registration assumes a correspondence of the original and deformation surfaces and conducts matching through a geometrical transformation. If the distance between the source and target point sets is greater than 2 cm, the stiffness of the object is reduced and the iterative improvement algorithm restarts optimization to obtain improved correspondence that provides better results.^5^, ^6^

### 2.2 Physical breast phantom

The correlation between the mean setup error as determined by the SGRT system and breast deformation was analyzed using physical breast phantoms. The shapes of the physical breast phantoms were designed using data from six patients from the radiotherapy department at Kanazawa University Hospital. All data in this study were from breast cancer patients treated to the lumpectomy because radiotherapy treatment can cause skin deformation.^9^, ^10^ The Institutional Review Board approved this retrospective study, which did not impact the rights or welfare of the patients (IRB number : 2019‐185). Three patient cases were used in investigation and three in validation. We knew the breast volumes, as delineated by radiation oncologists, from the treatment plans of the six patients. The investigated breast volumes of 300, 445, and 1315 cm^3^ were, respectively, considered to be representative of small, medium, and large breasts. Likewise, the validation breast volumes of 340, 435, and 750 cm^3^ were, respectively, considered to be representative of small, medium, and large breasts. The assigning of breast size followed the baseline of the breast tissue volume median in Japanese mammography examinations.^15^

**FIGURE 1:**
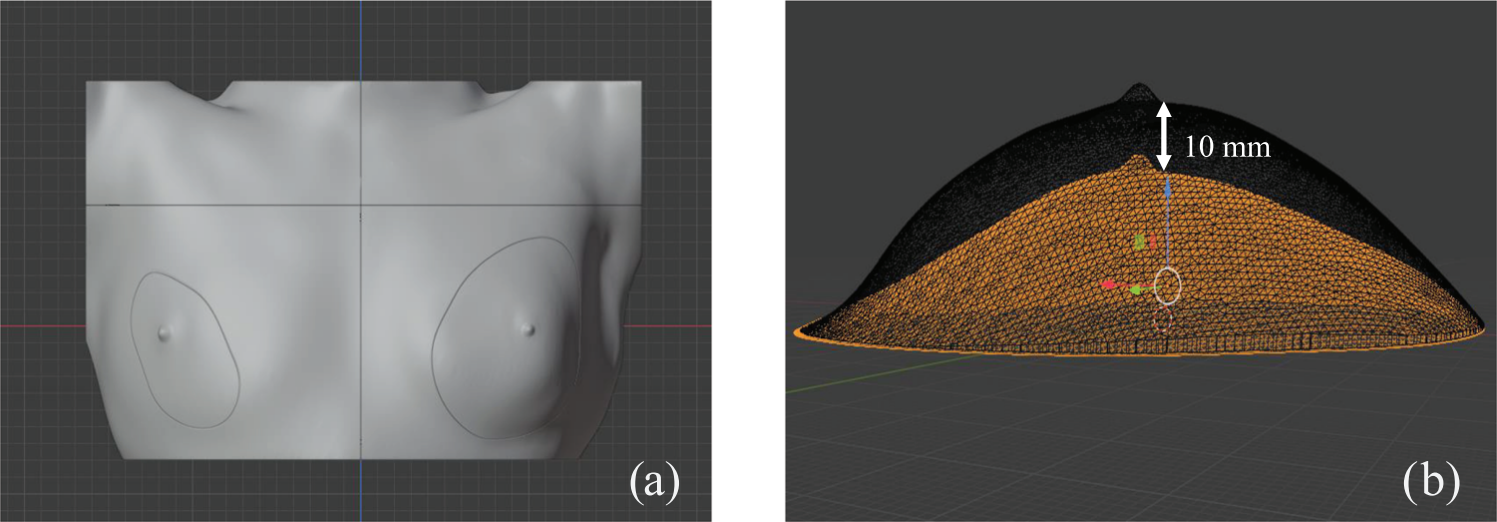
Standard size of physical breast phantom for validation in Blender: (a) whole part and (b) original breast part (orange) and extended breast part to 10 mm (black)

To make the physical breast phantom, we imported a CT DICOM file of the patient data to 3D Slicer (version 4.11.2), which is open‐source software for medical image processing. We created a three‐dimensional (3D) model of the patient outline from a raw CT image using the segment editor module and exported it into Blender (version 2.8.3). We then separated the body and breast part. The breast volume was not exactly the volume from the treatment plan because the junction between the breast part and body part should lie in the same plane for close alignment when we replace the original breast part with the deformed breast part (Figure 1a).

**FIGURE 2:**
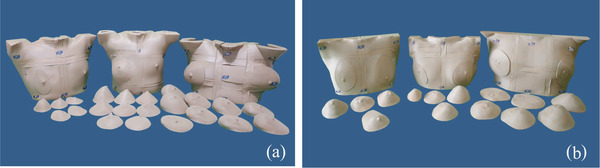
Physical breast phantoms with deformable breast parts: (a) three breast phantoms for investigation and (b) three breast phantoms for validation

The magnitude of breast deformation ranged from −30 to 30 mm. In the investigated cases, we deformed the ipsilateral side by −30, −15, −6, −3, 0, 3, 6, 9, 15, and 30 mm using software. The investigated small breast was not large enough to shrink and was thus only extended to 30 mm. The medium breast was shrunk by 15 mm and extended to 30 mm. The large breast was shrunk and extended to 30 mm. We therefore collected 25 datasets for investigation, including six datasets for the small breast, nine datasets for the medium breast, and 10 datasets for the large breast. In validation, both sides of the breasts were deformed in the range of ±30 mm; that is, by 0, 10, and 30 mm for the small and medium breasts and by −20, −10, 0, 10, and 30 mm for the large breast. There were thus 20 validation datasets, including six datasets for the small breast, six datasets for the medium breast, and eight datasets for the large breast. The extension and shrinking range of breasts is from −6 to 15 mm in a clinical situation^10^ but was widened to ±30 mm for the range test. We designed the direction of breast deformation based on research about breast deformation in patients during radiotherapy.^9^, ^10^ Breasts were deformed using the smooth proportional edit mode in Blender. We selected an area for vertical deformation. However, it is noted that there was also a small deformation effect in the surrounding area (Figure 1b). The data for all parts were exported to a da Vinci 1.0 Pro 3D printer (XYZ Printing, Taiwan). The printing material was a filament of polylactic acid. The physical breast phantoms for investigation (Figure 2a) and validation (Figure 2b) were sprayed with paint to give a skin tone color. The deformation difference of the breast piece between the Blender and the actual size was within 3 mm. Lead balls as CT imaging markers were placed at six points, namely two points in the mid‐section and four points on the two lateral sides at the top and bottom.

### 2.3 Data acquisition

We quantified the deformation of physical breast phantoms by comparing the surfaces of the original and deformed breasts. Each combination of the body part and breast parts was scanned with an Aquilion LB CT scanner (Canon Medical, Tokyo, Japan). The scan parameter settings were a slice thickness of 2 mm, tube voltage of 120 kV, and tube current exposure time of 300 mA s. The size of the physical breast phantom was slightly reduced by printing with the 3D printer. Therefore, the contour of each original physical breast phantom from the 3D printer in the CT images was used as a reference surface for positioning correction with the SGRT system. After we contoured each original physical breast phantom, the CT images with the phantom contour were transferred to a Monaco treatment planning system (Elekta AB, Stockholm, Sweden). The isocenter was placed on the chest wall, near the center of the base of the breast, below the junction of the body and breast part to achieve a similar isocenter for each test. We then transferred the planning data and reference surface to the linear accelerator and SGRT system.

**FIGURE 3:**
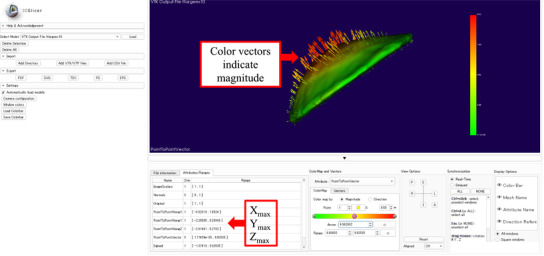
3D Slicer software displaying the three‐dimensional (3D) surface deformation and maximum values in *X*, *Y*, and *Z* directions for an extension of 10 mm deformation

The CT data for all physical breast phantoms, including the original and deformed phantoms, were imported to 3D Slicer for evaluation of the deformation. The segment editor module was used to define the region of interest as only the breast region manually. We then converted the region of interest to the label map volume using the segmentation module. The model‐to‐model distance module of 3D Slicer computed the distance between the reference and deformed surfaces. The source model (reference surface) was deformed to match the target model (deformed surface). If the deformed surface is the same as the reference surface, all vector lengths of the displacement vector field are zero and the displacement magnitude approaches zero. In contrast, the similarity between the two images decreases as the vector lengths increase from zero.^16^, ^17^ The mesh statistics module of 3D Slicer was used to obtain the maximum value in the field point‐to‐point along *X*, *Y*, and *Z* directions to analyze in which direction deformation most affects the mean setup error. The shape population viewer module was used to visualize and display the 3D surface deformation with scalars and vectors (Figure 3). The deformation of the breast part was evaluated using the maximum value in each direction according to

(1) $$D_{\mathrm{breast}}=\sqrt{X_{\max}^{2}+Y_{\max}^{2}+Z_{\max}^{2}},$$

where *D*_breast_ is the deformation of the breast part and *X*_max_, *Y*_max_, and *Z*_max_ are, respectively, the maximum deformations in *X*, *Y*, and *Z* directions.

**FIGURE 4:**
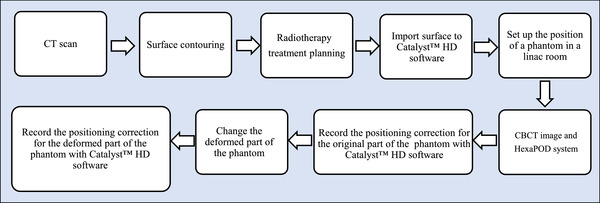
Workflow in collecting data of the difference between the original and deformed parts of physical breast phantoms

To assess the positioning accuracy of the SGRT system, the physical breast phantom was setup on a 6D treatment couch by matching the isocenter defined in the radiotherapy treatment plan to the isocenter defined in the linac room. In registration, an XVI CBCT system (Elekta AB) equipped on the linac acquired images to verify the phantom positioning setup. Additionally, the CT imaging markers on the phantom were used to check the registration of the CBCT image with the CT images of the treatment plan using an intensity‐based method with automatic and manual matching. The 6D treatment couch physically corrected the phantom positioning according to the result of the registration. The workflow for collecting the data from the SGRT system is shown in Figure 4. We used the masking tape to attach the body part of the physical breast phantom to the treatment couch for stability during changing the breast piece.

The optical camera settings of the SGRT system were an integration time of 4000 μs, gain of 350%, and surface averaging of 6 s, ensuring the same setup setting for the simulation of the patient. The surface tolerance setting was 10 mm and displayed in a submillimeter unit for position correction. We setup the boundary of the scan volume to fit the physical breast phantom for small/standard/large size, ensuring the same setting for consistency during collecting the datasets.

The original breast part was replaced with each of the deformed breast parts in assessing the cases of breast deformation. In the position correction, the contour of the physical breast phantom from radiotherapy treatment planning was used as a reference, and the surface image of the physical breast phantom on the treatment couch was taken as the actual image. The SGRT system calculated and displayed the six‐degree‐of‐freedom errors for correction. There were 25 datasets for investigation and 20 datasets for validation. We conducted the measurement three times at intervals of 10 s to account for instability and realized continual real‐time guidance using the SGRT system. The positioning setup error was represented as lateral, longitudinal, and vertical translational errors and pitch, roll, and yaw rotational errors. After we obtained the setup error for each dataset, we averaged the data to obtain the translation and rotation errors in each direction.

### 2.4 Data analysis

We compared the deformable and rigid registrations from analyzing tool in the Catalyst™ HD system. The performance of deformable registration was represented as mean absolute difference (MAD), according to

(2) $$\mathrm{MAD}\;=\frac{\sum_{i\;=\;1}^{n}\left|\mathrm{XD}-\mathrm{XR}\right|}{n}$$

where XD is the deformable registration value, XR is the rigid registration value, and *n* is amount of data.

The MAD was compared in lateral, longitudinal, and vertical translational errors and pitch, roll, and yaw rotational errors. The pair *t*‐test was used to determine statistically significant differences, and a *p*‐value less than 0.05 was considered to show statistical significance using SPSS version 20.0 (SPSS, Chicago, IL, USA).

The mean translation error obtained from measurement by the SGRT system (MT_measured_) and the mean rotation error obtained from measurement by the SGRT system (MR_measured_) were calculated as

(3) $${\mathrm{MT}}_{\mathrm{measured}}=\sqrt{\mathrm{la}t^{2}+\ln g^{2}+\mathrm{vr}t^{2},}$$

(4) $${\mathrm{MR}}_{\mathrm{measured}}=\sqrt{\mathrm{pitc}h^{2}+\mathrm{rol}l^{2}+\mathrm{ya}w^{2},}$$

where lat, lng, and vrt are, respectively, the lateral, longitudinal, and vertical translation errors and pitch, roll, and yaw are, respectively, the pitch, roll, and yaw angular errors.

We constructed a graph of the calculation of the mean translation error (MT_cal_), calculation of the mean rotation error (MR_cal_), and *D*_breast_ calculated using Equation (1). *X*_max_/*Y*_max_/*Z*_max_ and MT_measured_/MR_measured_ were used to plot a linear regression model. The regression coefficient of the linear regression model was adopted to find the weight factors of *X*_max_/*Y*_max_/*Z*_max_ for the calculation of MT_cal_/MR_cal_ using SPSS version 20.0.

The correlation between the *X*_max_/*Y*_max_/*Z*_max_ deformations and MT_measured_/MR_measured_ was expressed as a coefficient of regression (*R*), and a *p*‐value less than 0.05 was considered to show statistical significance. In testing the accuracy of the calculation of MT_cal_/MR_cal_, the correlations of MT_measured_/MR_measured_ and MT_cal_/MR_cal_ were analyzed using the coefficient of determination (*R* ^2^) from the scatter plot. We then used the linear trend of the calculations of MT_cal_/MR_cal_ to construct the graph of *D*_breast_ and MT_cal_/MR_cal_. We validated the equation using data obtained for the phantoms.

## 3 RESULTS

**FIGURE 5:**
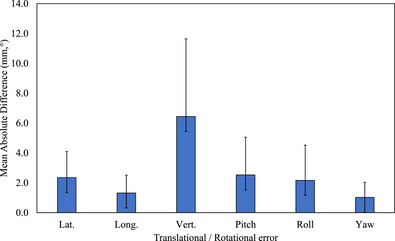
Mean absolute difference in lateral/longitudinal/vertical translational errors and pitch/roll/yaw rotational errors

Comparison of MAD for six directions had the most value with vertical. The MAD value of lateral/longitudinal/vertical/pitch/roll/yaw were, respectively, 2.4 ± 1.7 mm, 1.3 ± 1.2 mm, 6.4 ± 5.2 mm, 2.5° ± 2.5°, 2.2° ± 2.4°, and 1.0° ± 1.0°, showing statistical significance (*p* ≤ 0.05) except the longitudinal direction (*p* > 0.05) (Figure 5).

**FIGURE 6:**
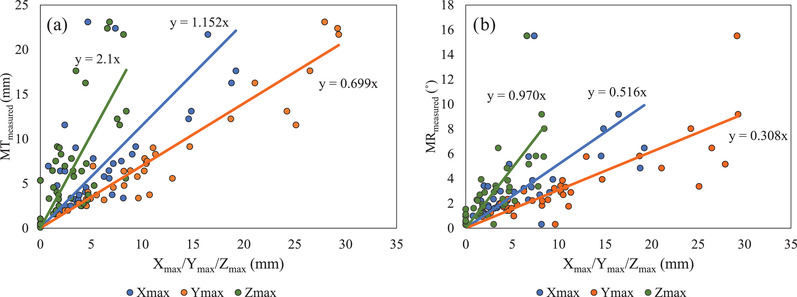
Correlations between *X*_max_/*Y*_max_/*Z*_max_ and (a) MT_measured_ and (b) MR_measured_

Analysis shows that MT_measured_ had the best correlation with *Y*_max_. The *R* values of the correlation between MT_measured_ and *X*_max_/*Y*_max_/*Z*_max_ were, respectively, 0.643, 0.949, and 0.719, showing statistical significance for all directions of deformation (*p* ≤ 0.05). MR_measured_ also had the best correlation with *Y*_max_. The *R* values of the correlation between MR_measured_ and *X*_max_/*Y*_max_/*Z*_max_ were, respectively, 0.586, 0.832, and 0.711, showing statistical significance for all directions of deformation (*p* ≤ 0.05) (Figure 6).

Equations for MT_cal_/MR_cal_ are obtained using the weight factors of *X*_max_/*Y*_max_ /*Z*_max_ obtained from linear regression analysis:

(5) $$MT_{\mathrm{cal}}=-0.226X_{\max}+0.794Y_{\max}-0.289Z_{\max}+0.934,$$

(6) $${\mathrm{MR}}_{\mathrm{cal}}=-0.029X_{\max}+0.282Y_{\max}-0.076Z_{\max}+0.319,$$

**FIGURE 7:**
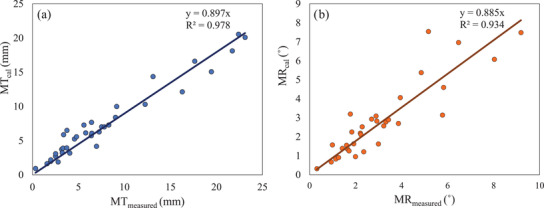
Correlations of (a) MT_measured_ and MT_cal_ and (b) MR_measured_ and MR_cal_

The *R* ^2^ values of the correlation of MT_measured_/MR_measured_ and MT_cal_/MR_cal_ were 0.978 and 0.934, respectively (Figure 7). These *R* ^2^ values were close to 1, and the calculated data thus correlated strongly with the measurement data of the SGRT system. These results confirm that it is possible to predict MT_cal_/MR_cal_ using the above equations.

We use the linear trends of Equations (5) and (6) to obtain the relations between MT_cal_/MR_cal_ and *D*_breast_:

(7) $$MT_{\mathrm{cal}}=0.521D_{\mathrm{breast}},$$

(8) $$MR_{\mathrm{cal}}=0.216D_{\mathrm{breast}},$$

where *D*_breast_ is the deformation of the breast part from Equation (1).

In validation, the values of MT_measured_/MR _measured_ obtained for validation cases were close to MT_cal_/MR_cal_. Among the 20 validation datasets, the difference between MT_measured_ and MT_cal_ was within ±5 mm for 16 datasets and more than ±5 mm for four datasets. Among the four datasets having a difference between MT_measured_ and MT_cal_ greater than ±5 mm, one dataset was for the original breast size and the others were for *D*_breast_ greater than 29 mm. The difference between MR_measured_ and MR_cal_ was within ±5° for 17 datasets and more than ±5° for three datasets, all of which had *D*_breast_ greater than 30 mm.

## 4 DISCUSSION

**FIGURE 8:**
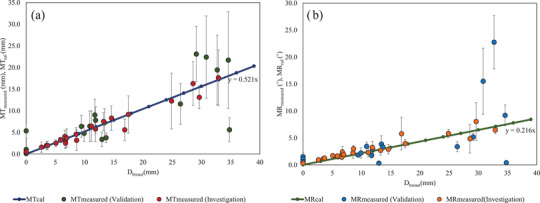
Correlations between (a) MT_measured_ (mm)/MT_cal_ (mm), (b) MR_measured_ (˚)/MR_cal_ (˚), and deformation of the breast part

Among the 45 datasets for the investigation and validation cases, the difference between MT_measured_ and MT_cal_ was within ±5 mm for 91% of the datasets and that between MR_measured_ and MR_cal_ was within ±5° for 93% of the datasets (Figure 8). The *R* ^2^ values of the correlation showed the calculated data was strongly correlated with the measurement data and had good accuracy within ±5 mm/±5°. Hence, our equations can estimate the mean translation and rotation errors in clinical situations. There were four datasets for which MT_measured_ obtained in validation cases exceeded ±5 mm. The one validation dataset for which the difference between MT_measured_ and MT_cal_ exceeded ±5 mm had MT_measured_ of 5.4 mm for the original breast size. The correction of the setup positioning of the original breast size should ideally be near zero. However, many factors, such as the uncertainty in the manual setup and registration due to the flexing of the physical breast phantom in the junction area, contributed to positioning setup error in this study. There were three datasets for which the difference between MT_measured_ and MT_cal_ exceeded ±5 mm and three datasets for which the difference between MR_measured_ and MR_cal_ exceeded ±5° when the deformation of the large breast exceeded 29 mm. The deformable registration restarted optimization when the distance between the source and the target point sets exceeded 2 cm.^5^, ^6^ This could be due to uncertainty arising in the Catalyst™ HD system. The uncertainty in the processing system used in this study was similar to that in the study of Walter et al.,^3^ who analyzed thoracic, abdominal, and pelvic regions with the Catalyst™ HD system. They found that the registration includes uncertainty because of the difficulty in detecting different shapes.^18^

**FIGURE 9:**
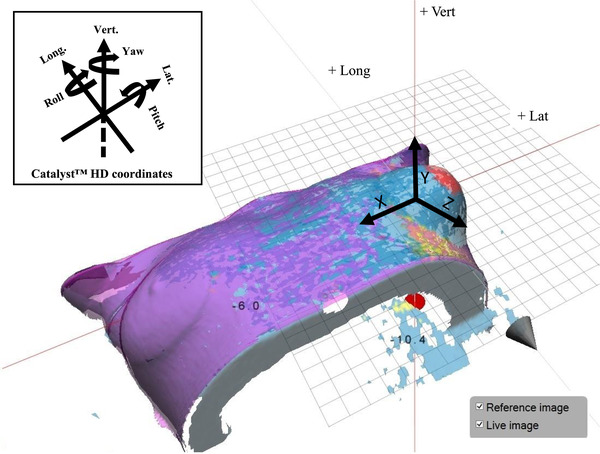
Catalyst™ HD system displaying a large breast part extended by 30 mm in the *Y* direction

The deformable algorithm of the Catalyst™ HD system using a simple physical breast phantom may show different magnitudes of effect of breast deformation on the mean translation and rotation errors. *D*_breast_ has linear correlations with MT_measured_/MR_measured_ ; that is, an increase in the magnitude of breast deformation increases MT_measured_/MR_measured_. The breast deformation relates to deformation in multiple directions but both MT_measured_ and MR_measured_ were most correlated with *Y*_max_. The performance of the deformable registration results shows the MAD had the most value in vertical direction when breast deformation. This could be due to breast deformation, which includes swelling and shrinkage, being largely in the vertical direction^9^, ^10^ (Figure 9). A comparison of our results with previously published results shows that we obtained the same result as Meyer et al.,^13^ who characterized the Catalyst™ HD system for breast size. A change in breast size is usually seen in the vertical direction because of the geometry of breast deformation. However, our results are slightly greater than their results because of the adoption of different methods of simulating breast deformation and different populations.

We derived appropriate equations for estimating the accuracy of the SGRT system for breast deformation. The equations were introduced to find the mean setup error when we know the maximum deformations in *X*, *Y*, and *Z* directions. The weight factors for constructing the equation are derived using the physical breast phantom. However, the weight factors used to construct an equation from the real breast deformation of a patient may be different.

Ono et al.^19^ calculated the setup margin for left‐sided breast radiotherapy during DIBH. They found the optimal planning target volume (PTV) margin as 3.59 mm from analyzing the systematic and random errors. This margin was useful for the setup of the breast cancer patients. Hence, the overall displacement tolerance should be within ±3.59 mm. In this study, we found that breast deformation of 7 mm affected the accuracy of the SGRT system with a tolerance of 3.59 mm. In practice, it is difficult to analyze maximum deformations in *X*, *Y*, and *Z* directions. However, the breast deformation can be estimated breast deformation in the *Y* direction from measurement on the CBCT image. The 2D vector magnitude of *Y* direction was defined by the maximum distance difference between the two registrations of the breast surface contour. The increase of magnitude in the *Y* direction leads to more position setup error. During a course of treatment, staff should take care when setting up a breast cancer patient because changes in the breast shape affect the mean setup error. After the patient setup was verified by CBCT imaging and the mean setup error exceeds 3.59 mm, the breast of the patient may have deformed. We can confirm by the magnitude in *Y* direction from CBCT images.

We represented breast deformation in terms of *X*_max_, *Y*_max_, and *Z*_max_. The minimum and average values in the *X*, *Y*, and *Z* directions are not close to the magnitudes of deformation created with the software in Section 2.2 whereas the maximum values are closer. However, there are various factors relating to breast deformation and the mean setup error, such as the appropriate selection of a reference surface,^13^ breast volume, posture of the patient, body mass index, and location of the breast treatment.^10^, ^20^

It is noted that there were limitations to the physical breast phantom because we designed the direction of deformation using software. The direction of deformation is more complex for a real patient than for the physical breast phantom and cannot be predicted. Therefore, the six cases examined in this study may not represent all clinical situations. In addition, we selected the area of deformation manually, which may introduce uncertainty into the evaluation of deformation when comparing the original and other breast sizes. One point to consider is that we cannot know exactly *X*_max_, *Y*_max_, and *Z*_max_ direction separately. However, the concept of this study can be adopted in testing the performance of other SGRT systems that can detect a deformed surface using a deformable model.

## 5 CONCLUSIONS

This study described the effects of the deformation of the breast surface on the positioning accuracy of radiotherapy and found that deformation occurs more in translation than in rotation. We found the magnitude of breast deformation affects the positioning accuracy of the Catalyst™ HD system. We derived an equation for estimating the error of the Catalyst™ HD system and found that the accuracy of the SGRT system is within 3.59 mm when the breast deformation is less than 7 mm. Additionally, when the breast of a breast cancer patient is deformed, the non‐rigid registration of the Catalyst™ HD system handle a large surface deformation that affects the setup.

## CONFLICT OF INTEREST

The authors declare no conflict of interest.

## AUTHOR CONTRIBUTIONS

Boriphat Kadman designed the experiments, made the phantoms, collected and analyzed data, and lead the writing of the manuscript. Akihiro Takemura supervised the work, collected and analyzed data, verified the analytical methods, provided critical feedback, discussed the results, and commented on the manuscript. Hironori Kojima collected data and aided in interpreting the results. Tatsuya Ito, Naoki Okada, and Shinichi Ueda collected data. All authors participated in writing the manuscript.
